# Differential expression and global analysis of miR156/*SQUAMOSA* promoter binding-like proteins (SPL) module in oat

**DOI:** 10.1038/s41598-024-60739-7

**Published:** 2024-04-30

**Authors:** Mehtab-Singh, Rajiv K. Tripathi, Wubishet A. Bekele, Nicholas A. Tinker, Jaswinder Singh

**Affiliations:** 1 Plant Science Department, McGill University, 21111 Rue Lakeshore, Montreal, QC H9X 3V9 Canada; 2 Ottawa Research and Development Centre, Agriculture and Agri-Food Canada, Ottawa, ON K1A 0C6 Canada

**Keywords:** Development, Flowering, Oat, Phase-transition, Reproduction, SPL/miR156, Functional genomics, Genomics, Plant development, Plant molecular biology, Plant reproduction

## Abstract

*SQUAMOSA* promoter binding-like proteins (SPLs) are important transcription factors that influence growth phase transition and reproduction in plants. *SPLs* are targeted by miR156 but the SPL/miR156 module is completely unknown in oat. We identified 28 oat *SPL* genes *(AsSPLs)* distributed across all 21 oat chromosomes except for 4C and 6D. The oat- *SPL* gene family represented six of eight *SPL* phylogenetic groups, with no *AsSPLs* in groups 3 and 7. A novel oat miR156 (AsmiR156) family with 21 precursors divided into 7 groups was characterized. A total of 16 *AsSPLs* were found to be targeted by AsmiR156. Intriguingly, *AsSPL3s* showed high transcript abundance during early inflorescence (GS-54), as compared to the lower abundance of AsmiR156, indicating their role in reproductive development. Unravelling the SPL/miR156 regulatory hub and alterations in expression patterns of *AsSPLs* could provide an essential toolbox for genetic improvement in the cultivated oat.

## Introduction

Cultivated oat (*Avena sativa L.;* 2n = 6x = 42, AACCDD) has a large (12.5 Gb) hexaploid (6x), repetitive genome with nonhomologous chromosomal exchange and functional divergence of duplicated genes that present significant challenges in the identification and characterization of novel genes^1^. Oat research has been accelerated recently with the release of a comprehensive annotated genome assembly that deciphered large-scale ancestral chromosome rearrangements including translocations and inversions^2^. The instability in the genomic structure of oats may be attributed to the absence of an orthologue equivalent to TaZIP4-B2 (located in the Ph1 locus) found in wheat. This orthologue in wheat plays a crucial role in stabilizing the genome structure by inhibiting crossing over among homeologous chromosomes during meiosis.^3–5^. Despite the aforementioned challenges, the recent availability of this high-quality genome assembly acted as a pivotal catalyst to pursue this study.

Transcription factors (TFs) are a huge group of regulators responsible for governing gene expression and act as on–off switches in controlling various developmental processes^6^. *SQUAMOSA* promoter binding like (SPL) proteins are plant-specific TFs responsible for the regulation of growth phase transitions including inflorescence development and architecture^7^. They have a conserved *SQUAMOSA* promoter binding protein domain (SBP) that comprises 76 amino acids^8^. The SBP domain binds to a sequence i.e., TNCGTACAA, where GTAC is the crucial core sequence found in the promoter region of the target gene^9^. The SBP domain consists of three essential motifs i.e., two zinc-binding fingers, Cys3His1 (Zn1) and Cys2HisCys (Zn2), and a bipartite nuclear localization signal (NLS) that moderately protrude over the Zn2 motif, which lies at the C-terminal of the domain^8,10^. So far, 16 *AtSPLs* in *Arabidopsis thaliana*^9^, 56 *TaSPLs* in wheat^11^, 19 *OsSPLs* in rice^12^, 17 *HvSPLs* in barley^13^ and 18 *BdSBPs* in *Brachypodium distachyon*^14^ have been reported. *SPL* genes regulate various developmental processes, including panicle architecture^15^, vegetative to reproductive phase change^16^, embryogenesis^17^, fertility^18^, hormonal signalling^19^, flowering^20^, copper homeostasis^21^ and germination and starch debranching^22^.

The advancements in the understanding of microRNAs (miRNAs) have introduced an additional layer of gene regulation. The miRNAs are a large group of small (20–24 nucleotides) non-coding RNAs that are initially reported to be involved in the larval differentiation of *Caenorhabditis elegans*^23^. Research findings suggest that plant miRNAs play a significant role in the gene regulation network of different developmental stages (review by Wang and Wang)^24^. MiRNAs target various transcriptional factor-encoding genes, making them a pivotal hub that regulates protein-DNA and protein–protein interactions^25^. More than half of the SPL transcriptional factors are targets of miRNA156, where antagonistic expression of *SPL* and miR156 have been observed^24^. In *Arabidopsis thaliana*, 10 of the 16 *AtSPLs* are targets of AtmiR156^20^, whilst 11 of 19 *OsSPLs* are targeted by OsmiR156 in rice^12^. MiR156 is involved in repressing flowering in *Populus canadensis*^26^, Chinese cabbage^27^, *Arabis alpina*^28^, and *Cardamine flexuosa*^29^. In rice, the overexpression of OsmiR156b delays flowering^12^, while the transition from the vegetative to the reproductive stage in barley is governed by the antagonistic expression of miR156 and *HvSPL* genes^13^. Despite various pathways (photoperiod, gibberellic acid [GA], autonomous, and vernalization) being reported for flowering regulation, the miR156/*SPL*s describe a new age-dependent pathway. We report the first comprehensive study of the *SPL*/miR156 regulatory hub in oat in which 28 *SPLs* (*AsSPLs*) and 21 novel precursors of miR156 (AsmiR156) were identified. This work includes phylogeny, gene structure analysis, conserved motifs, and AsmiR156 target site prediction. Furthermore, the differential expression of *AsSPLs *and AsmiR156 at different growth stages reveals their putative role in oat development. Elucidating the SPL/miR156 regulatory hub that governs reproductive transition in oat will aid in engineering heading date and improve oat adaptation to changing environments.

## Results

### Genome-wide analysis of *SQUOMOSA* promoter binding protein-like (*SPL*) gene family in oat

Based on similarity with the barley orthologs (*HvSPLs*) and the existence of the SBP domain, 28 putative *SPLs* were found in oat and were classified as *AsSPLs*. Twenty-five *SPL*s were identified in the latest *Avena sativa cv. Sang* genome *V1* and three additional *SPLs* (*AsSPL1D, AsSPL6D, AsSPL17D*) were identified in the *Avena sativa cv. OT3098 V2* genome. As expected, all *AsSPLs* had three copies in the hexaploid oat genome, except for *AsSPL23,* which had a single copy in the D genome. The genes were distributed across all 21 chromosomes except for chromosomes 4C and 6D. Interestingly, *AsSPL3s, AsSPL6s, AsSPL11s*, and *AsSPL15s* were not situated on homeologous chromosomes, likely due to ancestral chromosome rearrangements. The number of exons ranged from two to eleven, whilst the deduced length of the amino acid sequences varied between 179 and 1114. The majority of the *SPLs* were potentially localized in the nucleus, three in the chloroplast, two in the endoplasmic reticulum, and one each in vacuoles and extracellular regions (Table 1).

**Table 1:** Characterization of identified *SPL* genes in *Avena sativa.*

Gene^a^	Gene symbol^b^	CDS length^c^	Domain^d^	Deduced protein (aa)^e^	Ch^f^	Genomic position^g^	E value	Exon^h^	Predicted subcellular location^i^
*AsSPL1A*	AVESA.00010b.r2.3AG0429080	2130	SBP	710	3A	150,194,572–150,199,444	6.38e^−23^	11	Nucleus
*AsSPL1C*	AVESA.00010b.r2.3CG0485440	2592	SBP	864	3C	351,718,169–351,724,905	2.64e^−22^	11	Nucleus
AsSPL1D*	AVESA.00001b.r3.3Dg0000777	2643	SBP	881	3D	128,038,037–128,043,795	2.03e^−23^	11	Nucleus
*AsSPL3A*	AVESA.00010b.r2.1AG0030000	1659	SBP	553	1A	393,533,069–393,538,332	1.30e^−24^	5	Nucleus
*AsSPL3C*	AVESA.00010b.r2.6CG1116620	1149	SBP	383	6C	142,489,501–142,495,376	2.22e^−24^	3	Nucleus
*AsSPL3D*	AVESA.00010b.r2.2DG0387650	1647	SBP	549	2D	68,159,213–68,163,589	1.22e^−24^	5	Nucleus
*AsSPL6A*	AVESA.00010b.r2.4AG0617780	2082	SBP	694	4A	321,184,138–321,188,500	5.13e^−24^	9	Nucleus
*AsSPL6C*	AVESA.00010b.r2.7CG0713010	2238	SBP	746	7C	13,544,283–13,550,860	6.94e^−24^	10	Nucleus
AsSPL6D*	AVESA.00001b.r3.4Dg0002398	2871	SBP	956	4D	342,088,718–342,095,542	2.63e^−49^	11	Nucleus
*AsSPL9A*	AVESA.00010b.r2.1AG0053930	2553	SBP	851	1A	294,768,922–294,779,215	1.18e^−24^	10	Nucleus
*AsSPL9C*	AVESA.00010b.r2.1CG0079470	2697	SBP	899	1C	418,196,430–418,208,251	9.41e^−26^	11	Endoplasmic Reticulum
*AsSPL9D*	AVESA.00010b.r2.1DG0172650	2550	SBP	850	1D	276,378,031–276,388,313	1.39e^−25^	10	Endoplasmic reticulum
*AsSPL11A*	AVESA.00010b.r2.6AG1006610	735	SBP	245	6A	1,679,912–1,683,980	1.25e^−20^	3	Nucleus
*AsSPL11C*	AVESA.00010b.r2.6CG1110970	1005	SBP	335	6C	180,579,284–180,582,815	1.28e^−20^	4	Nucleus
*AsSPL11D*	AVESA.00010b.r2.2DG0382760	1005	SBP	335	2D	90,934,967–90,938,970	1.25e^−20^	4	Nucleus
*AsSPL13A*	AVESA.00010b.r2.2AG0198950	537	SBP	179	2A	24,988,625–24,991,456	2.64e^−18^	2	Nucleus
*AsSPL13C*	AVESA.00010b.r2.2CG0276270	546	SBP	182	2C	113,571,449–113,574,550	8.66e^−19^	2	Extracellular region
*AsSPL13D*	AVESA.00010b.r2.2DG0349760	537	SBP	179	2D	236,690,645–236,693,452	4.80e^−18^	2	Nucleus
*AsSPL15A*	AVESA.00010b.r2.7AG1218390	3318	SBP	1106	7A	134,554,545–134,560,310	1.03e^−27^	10	Chloroplast
*AsSPL15C*	AVESA.00010b.r2.5CG0931600	3342	SBP	1114	5C	15,625,436–15,630,899	1.54e^−27^	10	Vacuoles
*AsSPL15D*	AVESA.00010b.r2.7DG1390040	3318	SBP	1106	7D	49,217,131–49,222,850	4.47e^−25^	10	Chloroplast
*AsSPL16A*	AVESA.00010b.r2.5AG0853240	1317	SBP	439	5A	448,045,544–448,052,002	7.06e^−25^	3	Nucleus
*AsSPL16C*	AVESA.00010b.r2.5CG0880720	1299	SBP	433	5C	507,845,009–507,850,886	6.10e^−25^	3	Chloroplast
*AsSPL16D*	AVESA.00010b.r2.5DG0956210	1329	SBP	443	5D	401,441,491–401,447,497	5.07e^−25^	3	Nucleus
*AsSPL17A*	AVESA.00010b.r2.5AG0851230	1167	SBP	389	5A	441,621,396–441,625,091	6.81e^−21^	3	Nucleus
*AsSPL17C*	AVESA.00010b.r2.5CG0882850	1179	SBP	393	5C	499,731,050–499,735,457	4.78e^−22^	3	Nucleus
AsSPL17D*	AVESA.00001b.r3.5Dg0002304	1155	SBP	384	5D	399,247,804–399,251,340	2.16e^−23^	3	Nucleus
*AsSPL23D*	AVESA.00010b.r2.4DG0772040	1188	SBP	396	4D	337,532,631–337,537,680	1.18e^−13^	3	Nucleus

^a^Nomenclature of oat *SPLs* in this study.

^b^Gene accession number in the oat database.

^c^Length of coding sequence.

^d^Domain prediction by SMART tool.

^e^Number of amino acids in the protein sequence.

^f^Chromosomal location of *AsSPL* genes.

^g^Chromosomal coordinates of *AsSPL* genes in the oat genome.

^h^Number of exons in *AsSPL* genes.

^i^Predicted subcellular location of *AsSPL* genes.

*AsSPLs identified in the *Avena sativa cv. OT3098 V2* genome.

### Gene structure of *AsSPLs*

**Figure 1:**
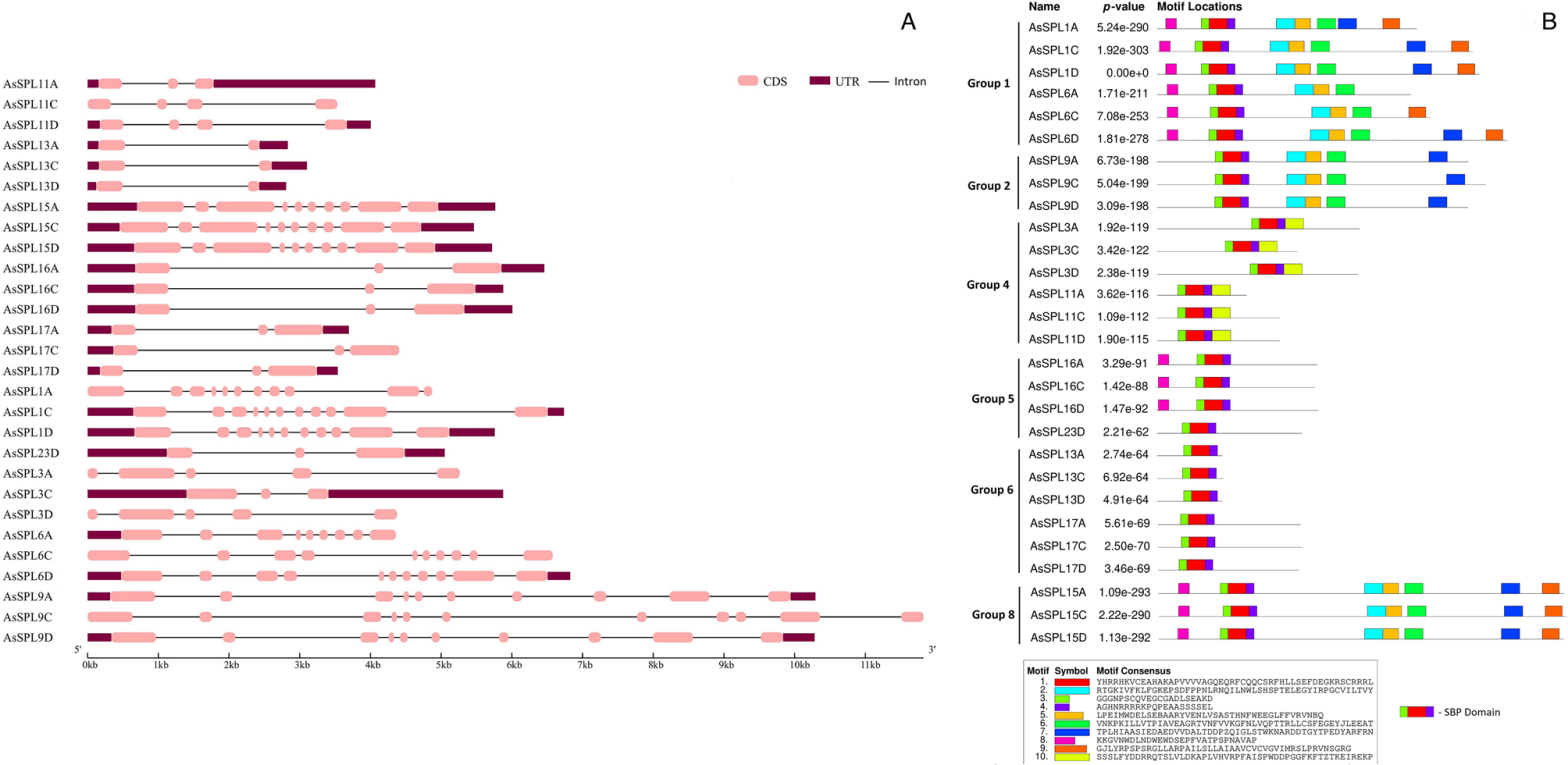
Gene structure of *AsSPLs* and conserved motifs in AsSPL protein sequences. (**A**) 5′ and 3′ UTRs are represented by dark red boxes; exons and introns are depicted using pink boxes and lines, respectively. The bar scale at the bottom corresponds to the gene size. (**B**) Distribution of conserved motifs in AsSPLs.

Examination of the genetic structure diversity of *AsSPLs*, which includes parameters like gene length, number, and distribution of exons and introns, revealed that at least one intron was found in the centre of the coding region of each *AsSPL*, and the number of exons in a gene ranged from 2 to 11 (Table 1). *AsSPL9C* had the longest gene size at 11.8 kb, while *AsSPL13D* had the shortest at 2.8 kb (Fig. 1A). The motif search in the *AsSPL* full-length protein sequences reported 10 conserved motifs. Motifs 1, 3, and 4 are present in each *AsSPL* and constitute the typical SBP domain; however, other motifs are undetermined (Fig. 1B). The *AsSPL13s, AsSPL17s,* and *AsSPL23D* contained no motif except those constituting the crucial SBP domain. As expected, the motifs were conserved amongst the homoeologues *AsSPLs*.

### Phylogenetic analysis and syntenic relationship of AsSPLs

**Figure 2:**
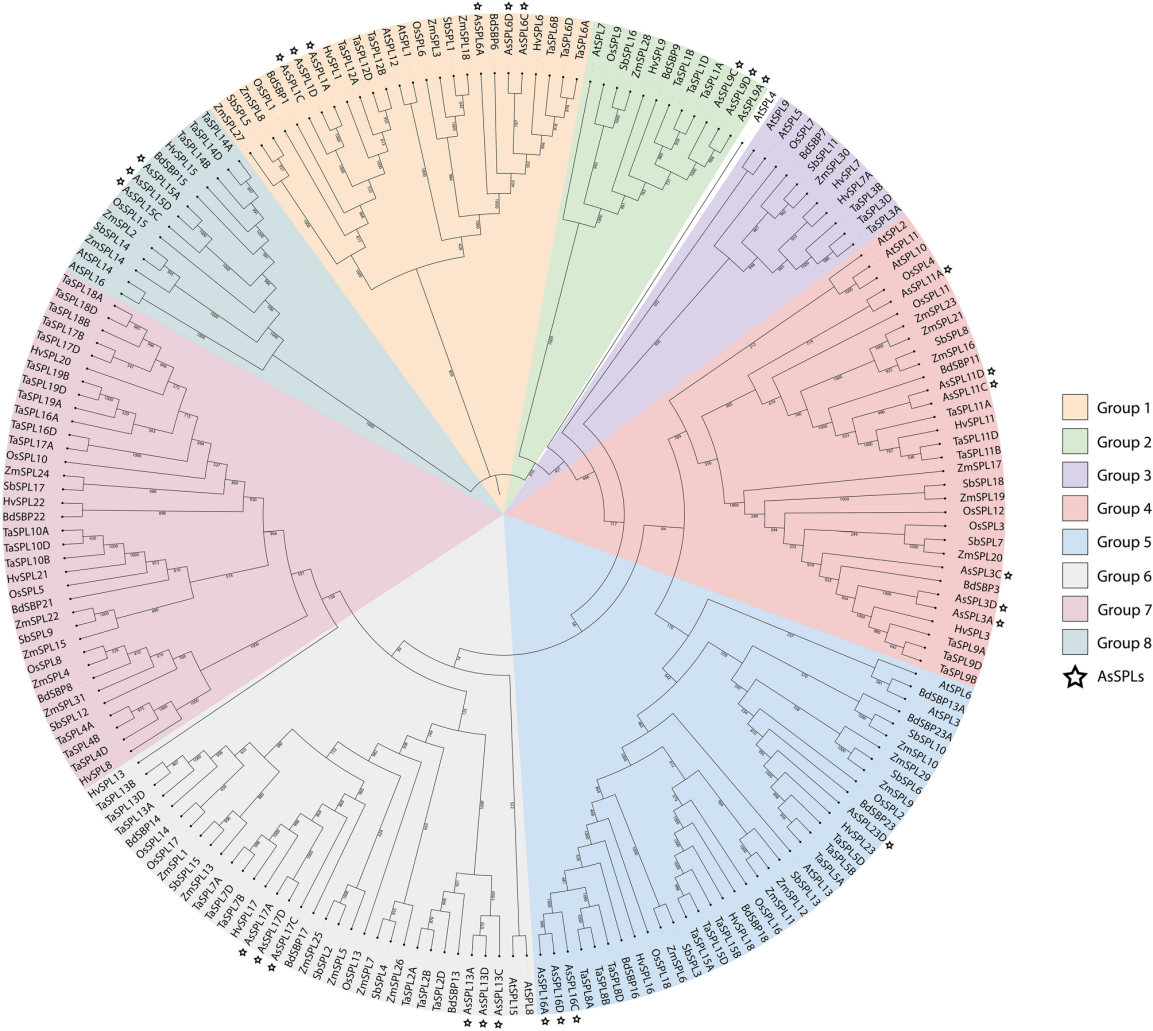
Phylogenetic analysis of SPL protein sequences. The neighbor-joining (NJ) tree of SPL proteins from *Arabidopsis thaliana* (AtSPL), rice (OsSPL), barely (HvSPL), wheat (TaSPL), sorghum (SbSPL), maize (ZmSPL), *Brachypodium distachyon* (BdSBP), and oat (AsSPLs). AsSPLs are highlighted using a star.

Characteristics of amino acid sequences and evolutionary relationships categorized SPL proteins were into eight groups with the *Arabidopsis thaliana* protein (AtSPL4) as an outlier (Fig. 2). The absence of any *AsSPL* members in group 3 and group 7 led to the division of the *AsSPL* gene family into 6 distinct subfamilies. The subfamilies with the smallest number of *AsSPL* members were Group 2 and Group 8, which had three members each, followed by Group 5, with four members, whereas the remaining subfamilies had six *AsSPLs* each. The oat *AsSPL11A* was closely related to the rice *OsSPL4* as compared to its homoeologues *AsSPL11C* and *AsSPL11D.*

**Figure 3:**
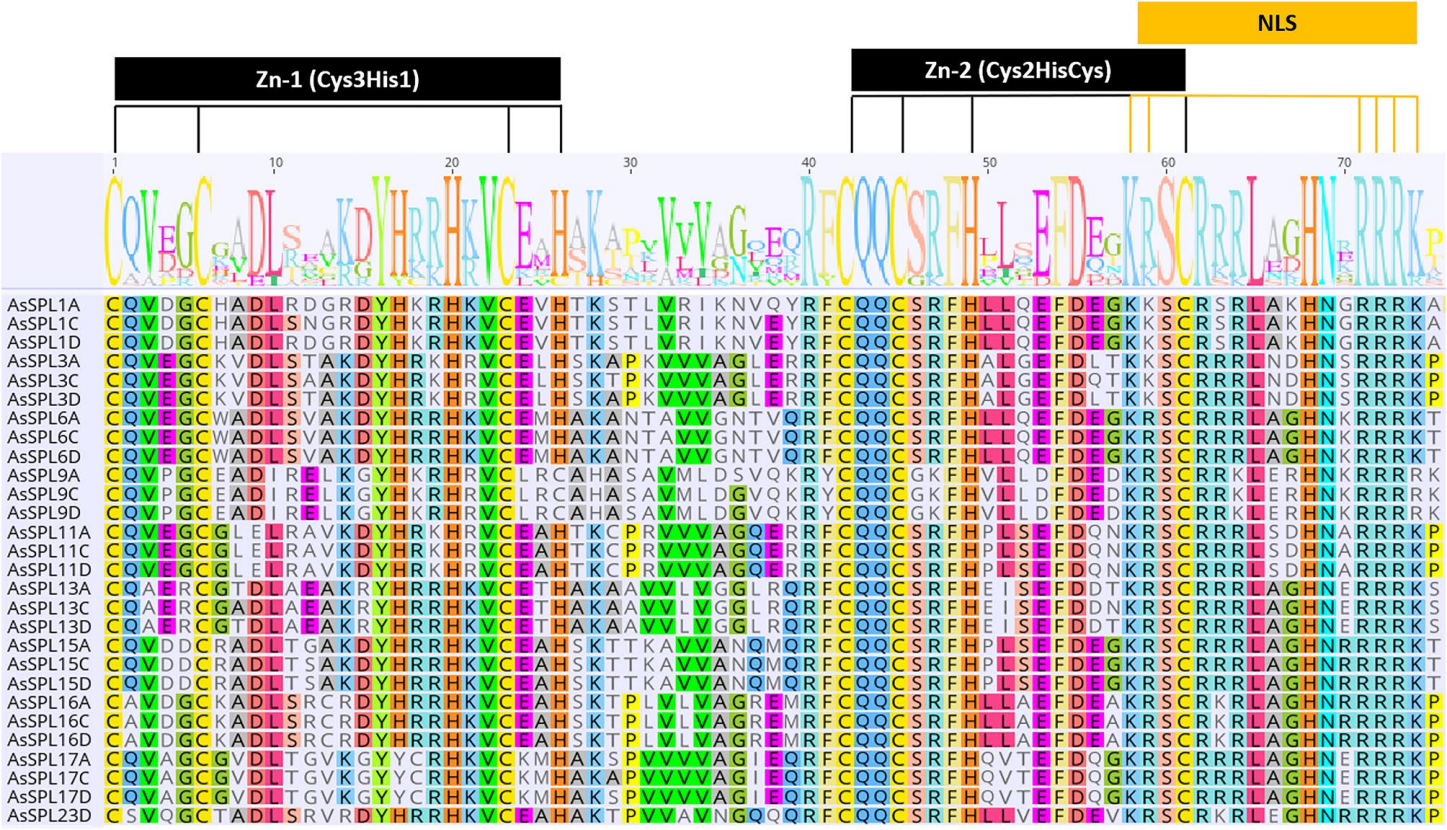
Multisequence alignment of SBP domain from AsSPL protein sequences. Two signature Zn fingers (Zn1, Cys3His1; Zn-2, Cys2HisCys) and one NLS region are marked on the top.

**Figure 4:**
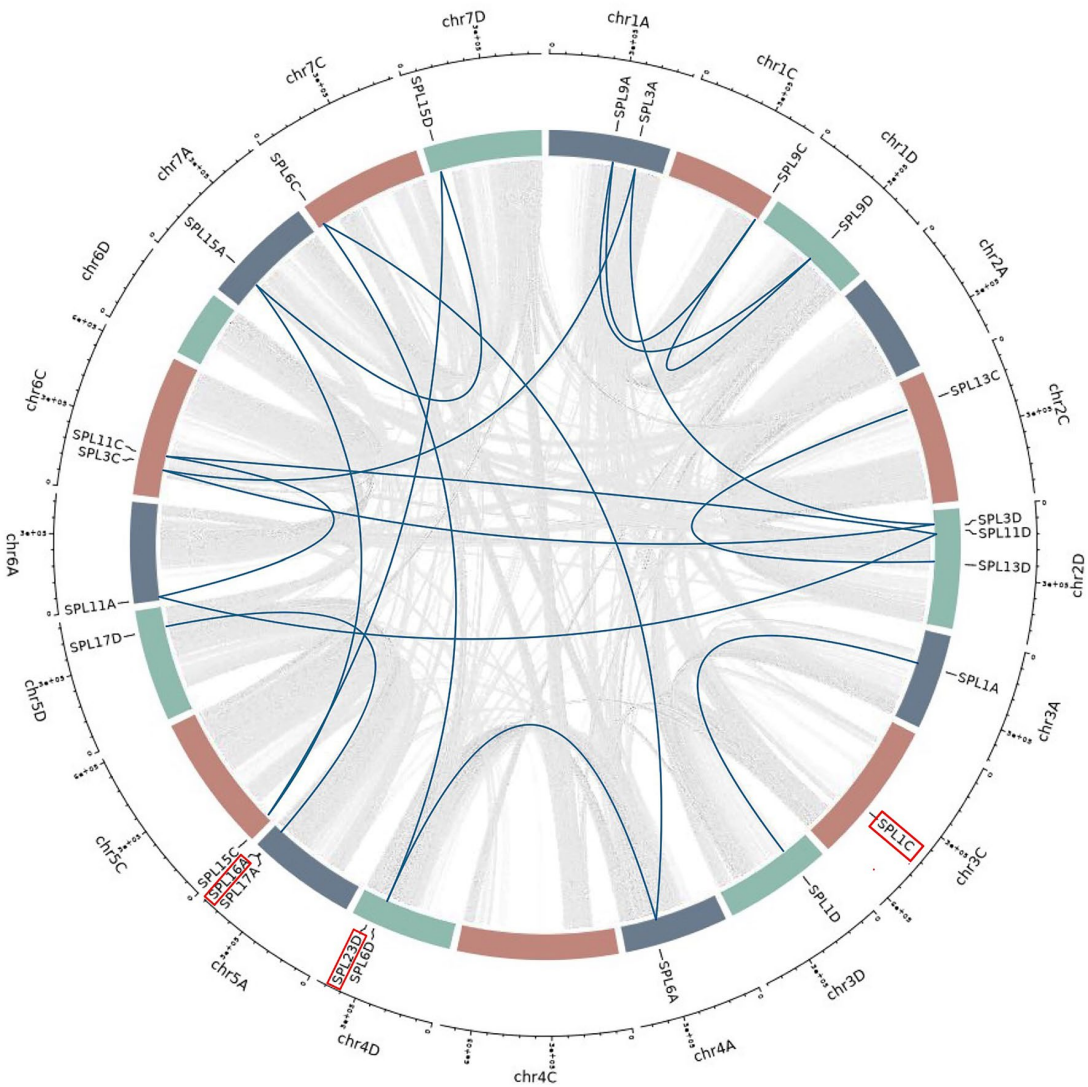
Synteny analysis of oat *SPL* sequences. The chromosomes on A, C, and D genomes are represented by blue, red, and green blocks, respectively. Syntenic relationships amongst all the annotated genes in the oat genome are depicted using grey lines, while synteny between the *AsSPLs* is shown using blue lines. Non syntenic *AsSPLs* are highlighted with a red box.

Two highly conserved zinc fingers (Zn-1 and Zn-2) and a nuclear localization signal (NLS) were found in the SBP domain (Fig. 3). Interestingly, *SPL9A/C/D* has a single amino acid mutation in the Zn-1(Cys-Cys-Cys-His), where the fourth histidine is mutated to cysteine. In addition to this, the oat SBP domain also displays significant conservation of specific sequences, namely CQQC, SCR, and RRR, suggesting their importance in the domain’s functionality. Synteny analysis of oat SPL proteins demonstrated synteny amongst most of the *AsSPLs* except *AsSPL1C, AsSPL16A,* and *AsSPL23D*, which were non-syntenic (Fig. 4).

### *The cis-*regulatory elements in *AsSPLs* promoter regions

The promoter region (1-kb upstream) of *AsSPL* genes contained 83 *cis-*acting elements (Table S1). These elements were further divided into seven groups, named development-related elements, light-response elements, environment stress-related elements, promoter-related elements, hormone-responsive elements, site-binding-related elements, and other elements. Amongst these, light-response-related elements (21) and other elements (22) covered a significant proportion, followed by development-related elements (12). Most *AsSPLs* were found to contain regulatory elements responsive to abscisic acid (ABRE) and methyl jasmonate (TGACG-motif and CGTCA-motif). However, a small number of genes contained regulatory elements related to other plant hormones, such as gibberellin (P-box, TATC-Box, and GAREmotif), auxin (AuxRR-core and TGA-element), ethylene (ERE), and salicylic acid (TCA-element). Light-response-related element: G-box was the most abundant, while Sp1 was identified in almost half of the *AsSPLs.* Amongst the development-related elements, a wide range of elements were found to be involved in the regulation of zein metabolism (O2-site), meristem (CAT-box and dOCT), endosperm (GCN4_motif, and AAGAA-motif), seed-specific (RY-element), root-specific (as-1, motif I), and phloem-specific (AC-I, II) expression. Four defence-related elements were identified in a few *AsSPLs,* including wounding and pathogen responsiveness (W box, box S, WUN-motif, and TC-rich repeats). Besides this, essential elements for anaerobic induction (ARE), anoxic-specific (GC-motif, and LTR) and drought inducibility (MBS) were widely distributed across the *AsSPLs.* The presence of transcription initiation related promoter elements (TATA-box, and CAAT-box) in all the *AsSPLs* validates the reliability of our promoter analysis. Finally, 22 other elements with unknown functions were also found out of which STRE and MYB are present in around 94% of the *AsSPLs.*

**Figure 5:**
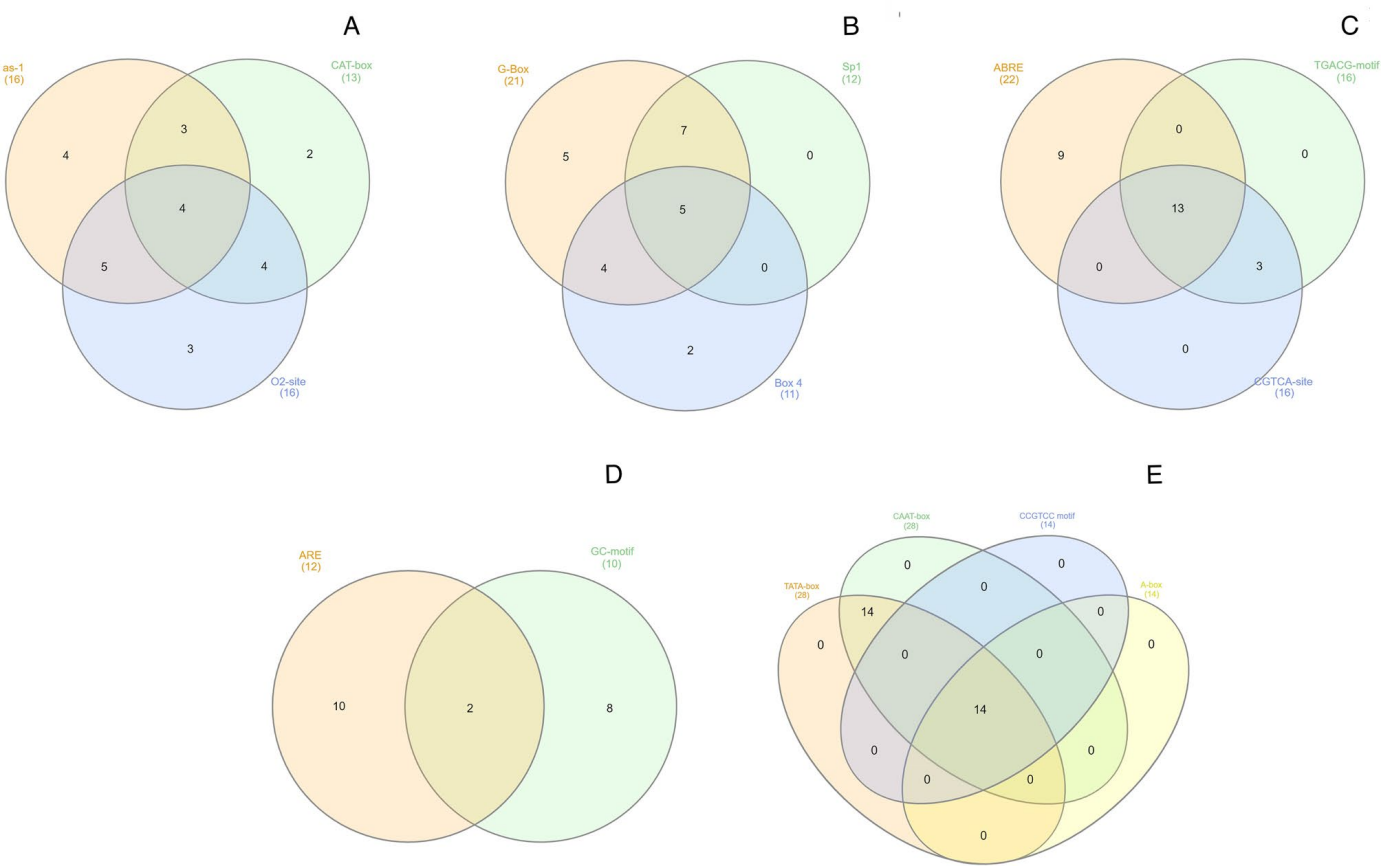
Venn analysis of cis-*acting* elements in the promoter regions of *AsSPLs*. (**A**) Development-related elements. (**B**) Light-responsive elements. (**C**) Hormone-responsive elements. (**D**) Environmental stress-related elements. (**E**) Promoter-related elements.

To gain a profound understanding of the importance of these cis-acting elements, a Venn diagram analysis was conducted specifically on those elements present in more than 10 genes (Fig. 5). A random distribution of light-responsive and development-related elements was observed among the *AsSPLs* (Fig. 5A,B). Amongst the hormone-responsive elements, 13 *AsSPLs* contained ABRE (ACGTG), TGACG-motif, and CGTCA-site at the same time (Fig. 5C). On the contrary, environment stress-related elements, i.e., ARE (AAACCA), and GC-motif (CCCCCG) were together present in only 2 *AsSPLs (AsSPL11C* and *AsSPL17A*) (Fig. 5D). Interestingly, out of 28 *AsSPLs*, 14 contained TATA-box and CAAT-box, while the other 14 had all four promoter-related elements, including CCGTCC motif and A-box (Fig. 5E).

### AsmiR156/*AsSPL* module in oat

**Figure 6:**
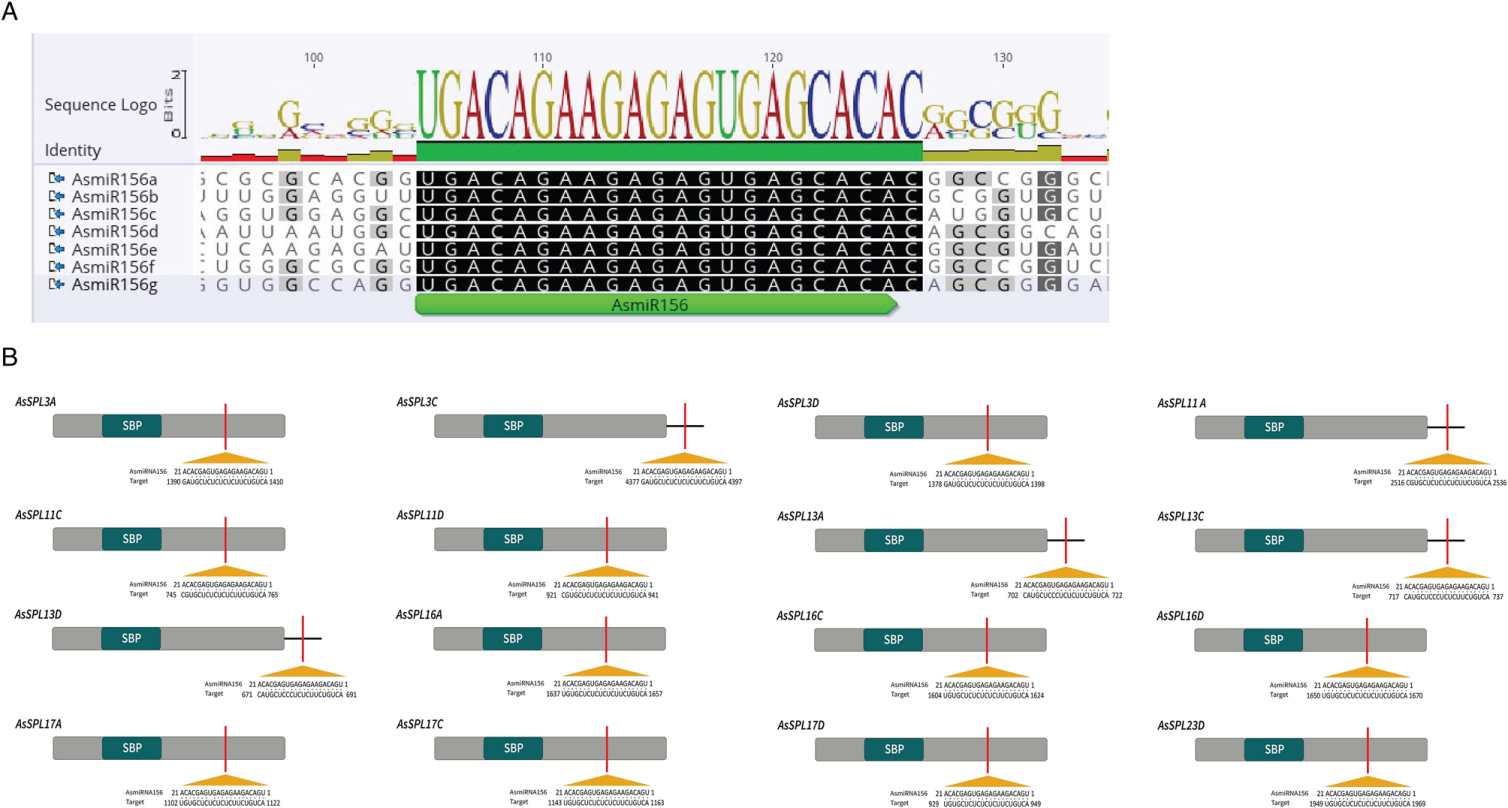
Oat miR156 (AsmiR156) family members and their target site in oat *AsSPL* genes. (**A**) Alignment of mature sequences of seven AsmiR156 family members. The mature sequence of AsmiR156 a/b/c/d/e/f and g are annotated in green. (**B**) miR156 target site in *AsSPL3s, 11 s, 13 s, 16 s, 17 s,* and *23* genes. The grey box represents CDS, green box SBP domain and line 3′UTR. The miR156 target sites with the nucleotide positions of *AsSPL* transcripts are marked in red. RNA sequence of each complementary site from 5′ to 3′ and the predicted miRNA sequence from 3′ to 5′ are indicated.

There is abundant evidence demonstrating a conserved role of miR156/*SPL* module in critical developmental processes in various crops^24^. However, there is no published report on miR156 in oat. Genome-wide investigation for miR156 precursors and mature sequences identified 21 putative AsmiR156 genomic loci. The length of precursors ranged from 170 to 264 nucleotides (Table 2 and Table S6), falling in the desirable range for plant miRNA precursors^30^. With few exceptions, the AsmiR156 precursors were found across all the oat chromosomes. As expected, the precursor sequences were distributed into 7 groups (*AsmiR156a-AsmiR156g*) of three highly conserved copies in each sub-genome (A, C, and D) (Fig. 6A). Intriguingly, group II and III precursors (*AsmiR156b and AsmiR156c*) on chromosome 3 A/C/D lie very close to each other (196–244 bp) and may act as a single polycistronic unit.

**Table 2:** Characteristics of putative *pre-miRNA156* in oat.

S. no	Group	Chr	miR156 Ids	Start	End	Str	LP	MFE (kcal/mol)	NM
1	I	2A	*AsmiR156a-A*	34,17,05,476	34,17,05,655	Reverse	180	− 86	2
2		2C	*AsmiR156a-C*	45,92,20,714	45,92,20,907	Reverse	194	− 101.8	2
3		2D	*AsmiR156a-D*	13,44,21,557	13,44,21,737	Reverse	181	− 93.8	3
4	II	3A	*AsmiR156b-A*	7,22,01,197	7,22,01,377	Reverse	181	− 68.6	1
5		3C	*AsmiR156b-C*	43,59,00,105	43,59,00,292	Forward	188	− 85.3	3
6		3D	*AsmiR156b-D*	2,95,36,326	2,95,36,523	Reverse	198	− 82.2	1
7	III	3A	*AsmiR156c-A*	7,22,01,601	7,22,01,805	Reverse	205	− 53.9	4
8		3C	*AsmiR156c-C*	43,58,99,669	43,58,99,861	Forward	193	− 76.1	2
9		3D	*AsmiR156c-D*	2,95,36,719	2,95,36,921	Reverse	203	− 64.5	2
10	IV	5A	*AsmiR156d-A*	41,71,67,598	41,71,67,802	Reverse	205	− 79.2	3
11		5C	*AsmiR156d-C*	45,37,82,523	45,37,82,730	Reverse	208	− 72	2
12		5D	*AsmiR156d-D*	37,11,50,225	37,11,50,437	Forward	213	− 88.1	3
13	V	6A	*AsmiR156e-A*	2,09,50,309	2,09,50,482	Reverse	174	− 69.7	2
14		6C	*AsmiR156e-C*	18,97,18,886	18,97,19,071	Reverse	186	− 70.1	2
15		2D	*AsmiR156e-D*	9,80,14,977	9,80,15,155	Reverse	179	− 76.2	2
16	VI	6A	*AsmiR156f-A*	28,33,67,292	28,33,67,471	Forward	180	− 90.5	3
17		6C	*AsmiR156f-C*	56,00,09,653	56,00,09,822	Forward	170	− 89.1	3
18		6D	*AsmiR156f-D*	24,00,58,412	24,00,58,591	Forward	180	− 91.6	3
19	VII	7A	*AsmiR156g-A*	19,45,68,262	19,45,68,444	Reverse	183	− 57.7	2
20		5C	*AsmiR156g-C*	11,18,83,176	11,18,83,398	Reverse	223	− 81.5	4
21		7D	*AsmiR156g-D*	10,50,94,730	10,50,94,993	Reverse	264	− 102.8	2

*Chr* chromosome, *Str* strand, *LP* length of pre-miRNA, *MFE* minimum fold energy, *NM* number of mismatches between predicted miRNA and miRNA.

AsmiR156 target site prediction identified 16 out of 28 *AsSPLs* as potential targets of AsmiR156 with putative binding sites in the CDS of eleven genes (Fig. 6B, Table S2). While, AsmiR156 targets five *AsSPLs* (*AsSPL3C, AsSPL11A, AsSPL13A, AsSPL13C, and AsSPL13D*) in the 3’UTR. In contrast to *AsSPL13s*, which exhibited 3' UTR target sites across all homoeologues, only the C and A genome copies of *AsSPL3s* and *AsSPL11s*, respectively, had an AsmiR156 target in the 3’UTR. These results indicate a probable post-transcriptional regulation of certain *AsSPLs* by AsmiR156, hence playing a key role in regulating the gene function in oat.

### Expression of *AsSPLs* and AsmiR156 during vegetative to reproductive transition in oat

**Figure 7:**
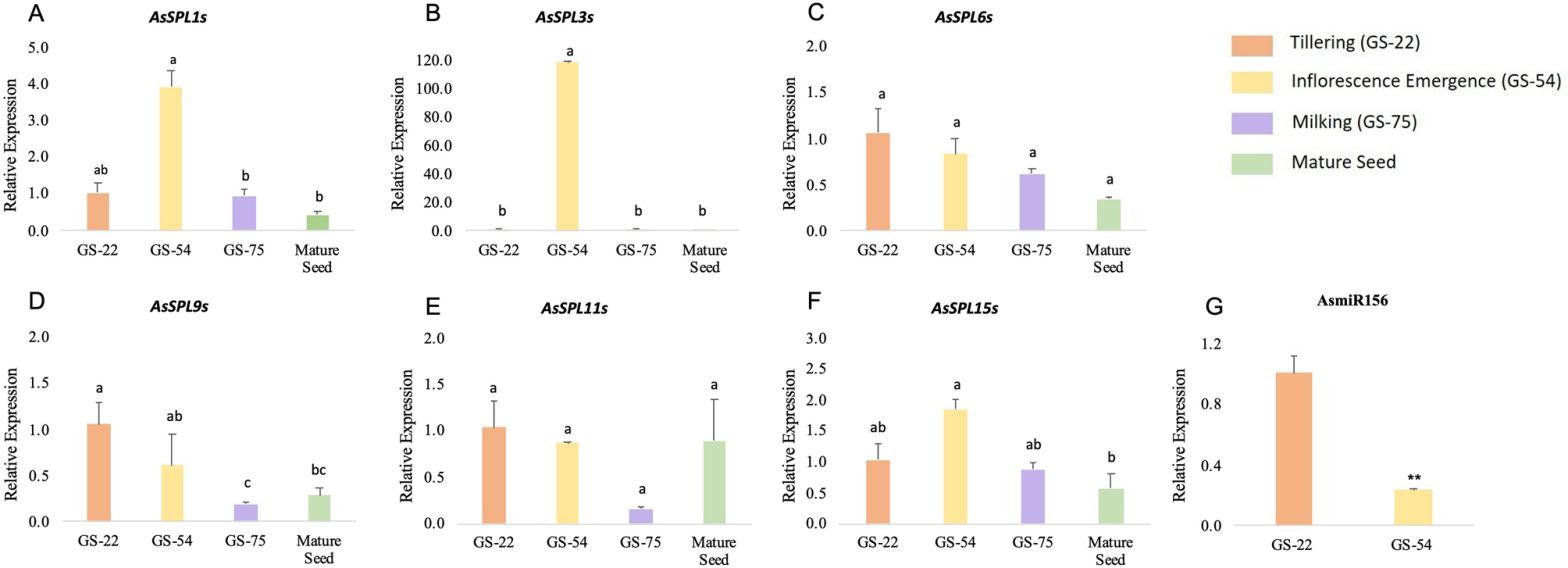
Expression pattern of *AsSPL* genes and AsmiR156 during growth phase transition in oat. (**A**–**F**) Transcript abundance of *AsSPL1s*, *AsSPL3s*, *AsSPL6s*, *AsSPL9s*, *AsSPL11*s and *AsSPL15s,* (**G**) AsmiR156. Error bars are indicated. The lowercase letter above the bar indicates the significant difference (P ≤ 0.05), while ** denotes (P < 0.01).

To gain an insight into the function of the *AsSPLs*, the transcript abundance of some *AsSPLs* and mature AsmiR156 was measured in oat at various growth stages using qRT-PCR. The stages include tillering (GS-22), inflorescence emergence (GS-54), milking (GS-75) and mature seed. The selection of *AsSPLs* was based on including members from different subgroups to ensure comprehensive coverage of gene diversity. A wide range of differential expression patterns were observed in the selected *AsSPLs* in different tissues (Fig. 7). A relatively high expression of *AsSPL9s* was observed at the vegetative stage, i.e., tillering (GS-22) (Fig. 7D). On the contrary, *AsSPL1s, AsSPL3s, and AsSPL15s* showed lower expression in the vegetative stage (GS-22) but higher in the developing inflorescence stage (GS-54) (Fig. 7A,B,F). Intriguingly, *AsSPL3s* had an extremely high expression (Fig. 7B) in the inflorescence emergence stage (GS-54) as compared to the vegetative tillering stage (GS-22). As expected, AsmiR156 abundance was higher during the vegetative stage (GS-22) which significantly reduced during the inflorescence emergence (GS-54) stage (Fig. 7G), implying the putative role of *AsSPL3s*/AsmiR156 in the vegetative to reproductive phase change in oat. The expression profiling was consistent in *AsSPL6s* and *AsSPL11s* across the different growth stages. Most of the selected *AsSPLs* showed comparatively low expression in the mature oat panicles.

## Discussion

This study presents the pioneer comprehensive genome-wide investigation of the *SPL*/miR156 hub in oat, identifying 28 oat *SPLs* (*AsSPLs*). The oat evolution process involving allohexaploidization has expanded the *AsSPL* gene family, leading to the formation of gene triplets. Polyploidy provides a fertile ground for gene duplication and neo-functionalization to occur. The additional gene copies resulting from whole genome duplications can undergo mutations and divergence, leading to the acquisition of new functions. This process has a significant role in the species’ evolution and contributes to their ability to acclimate to changing environments. The non-homoeologues distribution of *AsSPL3s, AsSPL6s, AsSPL11s*, and *AsSPL15s* can be attributed to various large-scale chromosomal rearrangements in the oat genome, affecting the order and distribution of these *AsSPLs* in the subgenomes (Fig. 4)^2^. Although different *SPL*s may possess variable intron and exon numbers, the first and second exon encodes the SBP domain in plants^31^.

The close association of genes in the phylogenetic tree can be used to anticipate gene functions. Considering the proximate phylogenetic association between *AsSPL11s* and *OsSPL4,* oat *SPL11s* might regulate grain size and grain yield^15^. Similarly, *AsSPL17A/D* shares the same phylogenetic group as *OsSPL14s* and may promote tillering and branching in oat^24^. The motif composition of members of each subgroup can support the phylogenetic characterization of AsSPL gene members. The motifs were conserved within the *AsSPL* homeologs and the members of the same phylogenetic subgroup, but considerable variability was seen amongst the different *AsSPL* subgroups, which highlights the functional diversity of *AsSPL* gene members (Figs. 1B and 2). For instance, motif 10 is unique to group 4 *AsSPLs* (*AsSPL3s* and *AsSPL11s*), which may confer a distinct role to these *AsSPLs* that need further investigation. These results are parallel to the *SPL* gene studies in wheat^32^, maize^33^, and soybean^34^. All the *AsSPLs* contained the signature SBP domain with highly conserved motifs, namely Zn-1 and Zn-2 fingers, a NLS region, CQQC, SCR, and RRR. These conserved elements were also seen in the SBP domain of *SPL* genes in various plant species^13,32,35,36^. A single amino acid replacement of the fourth His residue to Cys in the first Zn finger of *AsSPL9s* was found in all the members of subgroup 2, suggesting the presence of special Zn-1 finger in this group that includes *SPL* gene members from a wide range of species, i.e., *Arabidopsis thaliana,* maize, wheat, rice, sorghum, barley, and *Brachypodium distachyon* (Fig. S1). This mutation in the zinc finger binding site may confer a special role to *SPL9s*. A single mutation in the cysteine and histidine residues of zinc fingers can significantly affect the SPLs binding with the target gene due to large structural changes in the protein^8^. Similar His to Cys mutation has also been reported in foxtail millet^35^.

*Cis*-elements primarily regulate gene expression in organisms that help them acclimate to variable environmental conditions and stresses^37^. Hence, 1000 bp region upstream of the start codon of *AsSPLs* was critically examined, and numerous *cis*-regulatory elements were identified (Table S1). Most of the *AsSPLs* contained light-response-related elements (Sp1, Box 4, and G-box), indicating the involvement of oat *SPLs* in plant light-response pathways. The GCN4_motif (TGAGTCA) unique to *AsSPL11s,* and *AsSPL17s*, demonstrates their role in endosperm expression. Concomitantly, negative regulation of phloem expression is limited to two C genome *AsSPLs* (*AsSPL9C,* and *AsSPL15C*) controlled by AC-I, and AC-II elements. Besides this, the association of *AsSPLs* in abiotic and biotic stress-related responses can be attributed to the presence of TC-rich repeats, MBS (drought inducibility), and ARE (anaerobic inducibility) elements. An alfalfa study has also reported improved drought tolerance by miR156-mediated silencing of *SPL13*^38^.

Interestingly, a few *AsSPLs* had *cis-*regulatory elements for pathogen and wounding response i.e., W box and box S. WRKY transcription factors are widely known for binding to W box element found in the promoter of seven *AsSPLs*, which activates a dynamic chain of signalling via phosphorylation cascades or kinases^39^. *HvSPL23* was found to positively co-express with the receptor kinase gene, *HvWAK1*, indicating its potential upstream regulator^40^. Interestingly, most of the *AsSPLs* contain auxin- associated elements (AuxRR-core, TGA-box). Auxin is important for root regeneration, callus induction and plant growth. Three *SPLs* (*SPL2, SPL10,* and *SPL11*) have been reported to suppress the expression of AP2/ERFs directly, preventing the auxin buildup in the callus, indicating their role in the auxin synthesis pathway^41^. Gibberellic acid (GA) is an essential hormone for the vegetative to reproductive phase transition pathways involving the *SPL*/miR156 module^24^. *AsSPLs* contained three gibberellin-responsive elements, i.e., P-box (*AsSPL13A, AsSPL13D, AsSPL17C*), GARE-motif (*AsSPL16A*), and TATC-box (*AsSPL1C*) indicating their potential involvement in the flowering pathways.

The miR156/SPL module appears to have a conserved function of flowering regulation in plant species, where its overexpression leads to a delay in flowering time in rice^12^, maize^42^, tomato^43^, and *Arabidopsis thaliana*^44^. The present study reports 21 novel AsmiR156 precursors in oat, which were divided into seven subgroups (*AsmiR156 a-g*) based on the homology among the precursor sequences (Table 2). The whole genome duplication events like allohexaploidization have an apparent role in the expansion of the miR156 family in oat. The mature sequence of miR156 is conserved amongst the subgroups and other monocots like barley and wheat. However, the proximity (196–244 bp) of group II and group III precursors (*AsmiR156b-A/C/D* and *AsmiR156c-A/C/D*) on their respective genomes could indicate their polycistronic nature. According to various genome-wide analyses of various miRNA genes, numerous clustered miRNAs might undergo simultaneous transcription forming a single polycistronic unit^25,45,46^. In *Arabidopsis thaliana*, 10 of the 16 *AtSPLs* are targets of AtmiR156^20^, whilst 11 of 19 *OsSPLs* are targets of OsmiR156 in rice^12^. The target site prediction analysis in oat showed 16 out of 28 *AsSPLs* as targets of AsmiR156 (Fig. 6B). The majority of *AsSPLs* possess target sites in the coding region, while five *AsSPLs* are targeted in the 3’UTR. Especially, *AsSPL13s* have AsmiR156 target sites in the 3’UTR, which are parallel to *HvSPL13* and *BdSBP13* in barley and *Brachypodium distachyon*, respectively. This shows the miR156 target sites are conserved within the homologous genes in different species. Moreover, the differences in AsmiR156 and *AsSPL* target site sequences were mostly detected in the 14^th^, 20^th^, and 21^st^ nucleotides, as observed previously in barley^13^. Hence, these sites have been under immense selection pressure during the course of evolution, thereby highlighting the critical role of AsmiR156 in governing the expression of *AsSPLs* in oat.

During plant developmental phases, the expression of *SPL* genes is upregulated due to the decline in miR156 abundance^24^. A similar pattern has been observed in AsmiR156 targeted *AsSPL3s*, where its expression markedly increased during the inflorescence emergence stage (GS-54). Whereas, the downregulation of *AsSPL3s* during the vegetative stage (GS-22) can be attributed to the abundant AsmiR156, implying the mRNA cleavage (Fig. 7B,G). The barley *HvSPL3* and *Brachypodium distachyon BdSBP3* also expressed differentially between the vegetative and early reproductive stages^13,14^. Moreover, the knockout mutants of *OsSPL3* and *OsSPL4* lead to changes in the heading date, suggesting the conserved role of group 4 *SPLs* in the flowering^47^. Nevertheless, the expression of *AsSPLs* (*AsSPL6s, AsSPL15s, AsSPL9s*) not targeted by AsmiR156 remained almost consistent over the plant growth stages, validating the conserved role of *SPL*/miR156 module in inflorescence development and reproductive phase change in oat.

Detailed knowledge of molecular mechanisms that regulate panicle and spikelet development could aid in engineering superior novel architectures and higher yield potential. Rising temperature and climate change scenarios will likely affect pollen viability and fertilization which may reduce yield. Studying such mechanisms can ultimately allow deliberate engineering of flowering time to improve adaptation to changing environments. The current study elucidates the critical role of the miR156/*AsSPL* hub in developmental phase transitions and panicle development in oat.

## Materials and methods

### Identification of *SPL* genes in oat

The GrainGenes (https://wheat.pw.usda.gov/jb?data=/ggds/oat-ot3098v2-pepsico) database was used to obtain the coding (Table S4), genomic (Table S3), and protein sequences (Table S5) of oat *SPLs* (*AsSPLs*). The barley SBP domain (Pfam: PF03110) was used as a query to perform tBLASTn against the annotated PepsiCo OT3098 Hexaploid Oat v2 pseudomolecules (2021) and the latest Sang genome^2^. The SMART tool (http://smart.embl-heidelberg.de/) was used to verify the SBP domain in the SPL protein sequences and subcellular localization of SPL proteins was predicted using the WoLF PSORT tool (https://wolfpsort.hgc.jp/). The accession numbers for *AsSPL* genes were extracted and putative oat *SPLs* were named based on their evolutionary relationship with barley *SPLs*.

### Gene structure, phylogeny, and synteny of *AsSPL* genes

The exonic and intronic regions of each *AsSPL* gene were obtained using the Gene Structure Display Server program (http://gsds.gao-lab.org/) by comparing their genomic and coding sequences. The *SPL* sequences of *Arabidopsis thaliana* were obtained from TAIR (https://www.arabidopsis.org/), whilst the *SPL* sequences of wheat, barley*, Brachypodium distachyon,* maize and sorghum were obtained from 1113 to 1452, respectively. The complete *SPL* protein sequences were aligned using MUSCLE, and a neighbor-joining (NJ) phylogenetic tree was constructed using MEGAv11.0 by setting a bootstrap value of 1000. Further annotation of the phylogenetic tree was performed in iTOL (https://itol.embl.de/). The syntenic relationships amongst the *AsSPLs* were evaluated using the MCScanX^49^ and visualized using the shinyCircos-V2.0^50^.

### MiRNA156 family in oat and their target site prediction in *AsSPLs*

Previously reported Hv-miR156 sequence in barley was retrieved from^13^ to conduct a homology-based search. For short query sequences, the BLASTN algorithm was employed with an increased e-value (E = 10) in the Geneious Prime Software (https://www.geneious.com) for comparison of reference HvmiRNA against the *Avena sativa cv. Sang* genome. Matches that exhibited a similarity of at least 95% were selected for further analysis. Around 80–250 bp upstream to 80–250 bp downstream regions of the mature miRNA were extracted to obtain pre-miRNA sequences^51^. M fold software was used to predict fold-back secondary structures for pre-miRNAs^52^. The pre-miRNA structures were selected based on the criteria by Lu and Yang (2010). psRNATarget tool (http://plantgrn.noble.org/psRNATarget/?function) was used to anticipate the AsmiR156 target sites in cDNA sequences of *AsSPLs*.

### Identification of conserved motifs and *Cis*-acting elements

The conserved motifs in the AsSPL proteins were identified using default settings in MEME 5.4.1 (http://meme-suite.org/tools/meme), while the maximum width was adjusted to 50, the minimum to 6, and motifs were searched to a maximum number of 10. The Geneious Prime software was used for the creation of the sequence logo of the oat SBP domain sequences (https://www.geneious.com). The 1000 bp upstream sequences (promoter region) of the *AsSPLs* coding regions were searched for *cis-*regulatory elements using the PlantCARE database^54^. Further Venn analysis was carried out for the conserved *cis-*acting elements using InteractiVenn web-based software^55^.

### Plant material, sample preparation and RNA extraction

The oat cultivar Park obtained from PGRC, Saskatoon, Canada was planted in the growth chambers at Macdonald Campus, McGill University. The plants were grown with a 16:8 photoperiod ratio at day and night temperatures of 22 °C and 15 °C, respectively. A 20:20:20 (nitrogen: phosphorus: potassium) fertilizer was applied after sowing and at the tillering stage to promote plant growth. The young leaf (GS-22), immature panicles (GS-54, GS-75), and mature panicle samples from the oat plants were collected and immediately flash-frozen in liquid nitrogen before storing the samples at − 80 °C. Total RNA was extracted using the modified SDS-LiCl as described in^56^. Following this, RNA was quantified using a NanoDrop ND-1000 (NanoDrop Technologies, Wilmington, DE, USA), and gel electrophoresis was performed to check the purity and integrity.

### DNAse I digestion, cDNA synthesis, and quantitative real-time PCR (qRT-PCR)

DNA contamination was removed by DNase I treatment of all samples (Promega, USA). For each sample, 15 min of incubation at 23 °C, followed by the addition of 1 µl 25 mM EDTA to every sample, and the final incubation at 65 °C was performed for 10 min to terminate the reaction. From each sample, 500 ng of RNA was taken to synthesize cDNA using the AffinityScript QPCR cDNA Synthesis Kit (Agilent technology, Canada). For the amplification of miR156, stem-loop RT primer was used for cDNA synthesis. Due to high sequence similarity amongst the protein sequences of *AsSPLs* homoeologues, common primers for each gene were designed from the coding region ^57^. AsmiR156 was amplified using the AsmiR156 specific forward primer and universal reverse primer (Table S7). Optical strip tubes were used to perform qRT-PCR analysis using the Mx3000 qPCR system (Stratagene, USA) using 1 μl diluted cDNA, 10 μM gene-specific primers, and 10 μl Brilliant III Ultra-Fast SYBR® Green QPCR Master Mix (Agilent, USA). The above reaction was carried out by running it for 10 min at 95 °C, followed by 40 cycles of 15 s at 95 °C, and 30 s at 60 °C. Internal controls included the expression of a reference gene *EF1A*, which was recommended as the most consistent housekeeping gene amongst different growth stages in oat^58^. Relative gene expression was determined using the 2 − ΔΔCq method^59^. qRT-PCR was performed in duplicates and triplicates for *AsSPLs* and AsmiR156, respectively.

### Statistical analysis

One-way analysis of variance (ANOVA) and Tukey's test (P ≤ 0.05) were carried out using JMP Pro 16 software to determine statistical significance amongst the *AsSPL* expression at different growth stages of oat.

### Ethical approval

We confirm that the present research adheres to applicable institutional, national, and international standards and regulations for conducting plant experiments, including the collection of plant material.

### Supplementary Information

Supplementary Information.

## Data Availability

The data that support the findings of this study are available in the supporting materials of this article.
